# Dynamic Similarity Theory Based on Geometric Distortion and Material Compensation for On-Orbit Assembled Space Rod Structures

**DOI:** 10.3390/ma19183878

**Published:** 2026-09-11

**Authors:** Yongbo Ye, Sicheng Wang, Jianfei Yang, Dayu Zhang, Xiaofei Ma

**Affiliations:** China Academy of Space Technology (Xi’an), Xi’an 710100, China

**Keywords:** on-orbit assembly, geometric distortion, material compensation, flexible rods, scaled-down equivalent, dynamic similarity

## Abstract

On-orbit assembly technology is the core method for constructing extremely large space structures, with assembly modules serving as the fundamental units for structural integration. Since ground dynamic verification of full-scale modules is often restricted by laboratory space, scaled models are required for equivalent evaluation. During the scaling of systems containing high-aspect-ratio flexible rods, traditional complete geometric similarity laws lead to severe dynamic distortion in these slender elements as dimensions are reduced. To address this issue, a dynamic equivalence method based on geometric distortion and material compensation specifically for space flexible rods is proposed. This theory permits non-proportional distortion of rod cross-sections by deriving distortion similarity laws and reconstructs dynamic consistency through material substitution. Numerical validation demonstrates that the method effectively eliminates prediction errors induced by size reduction. For free single rods, the prediction errors for the first three bending frequencies are maintained within 1%; for unconstrained two-bar mechanisms connected by spatial spherical joints, the error is maintained within 0.5%. Furthermore, upon introducing sliding rail boundary constraints, the scaled model accurately reproduces the spatial mode shapes of the prototype, with primary frequency errors converging to within 0.3%. This research provides a reliable theoretical basis for the ground experimental evaluation of on-orbit assembly equipment for extremely large space structures.

## 1. Introduction

On-orbit assembly represents a disruptive technological paradigm for the realization of ultra-large space assets, including space solar power stations, massive satellite antennas, and large-aperture radio telescopes [1]. Distinct from conventional deployable structures, ultra-large space structures are characterized by high flexibility, low natural frequencies, and highly time-varying configurations during the assembly process. Given that the dimensions of such structures can reach the hundred- or even thousand-meter scale upon completion, the implementation of full-scale ground testing is practically precluded by engineering limitations. Consequently, the development of high-fidelity ground-based scaled models and the establishment of dynamic equivalent mapping between scaled units and full-scale prototypes have become essential for validating the on-orbit mechanical performance of large antennas [2,3,4,5,6]. In recent years, leading international space initiatives, such as NASA’s OSAM-1 mission and ESA’s on-orbit servicing explorations, have explicitly underscored that cross-scale ground equivalent testing is indispensable for mitigating on-orbit technical risks [7,8]. The current scientific challenge remains: how to bridge the massive scale gap and construct a rigorous dynamic and deformation scaling framework to achieve precise predictions of full-scale dynamic behavior using ground-based scaled prototypes.

Dynamic modeling and characterization of large space structures during the on-orbit growth phase are exceptionally challenging due to the massive number of modules and evolving topological relationships. Currently, research on on-orbit assembly dynamics primarily focuses on multibody system evolution and issues related to time-varying stiffness. To address large deformations and rigid-flexible coupling effects in flexible multibody systems, nonlinear dynamic modeling approaches, such as the Absolute Nodal Coordinate Formulation (ANCF), have demonstrated significant advantages and are widely integrated into the dynamic description of complex space structures [9,10]. On this basis, Li et al. [11] developed a variable-configuration rigid-body attitude–orbit coupled modeling method based on the natural coordinate formulation to address the dynamic challenges of on-orbit assembly of ultra-large space structures, investigating the dynamic evolution mechanisms and dominant disturbance torque characteristics during repeated assembly–deployment cycles. Wu et al. [12] and Fan et al. [13] proposed a dimension-reduction modeling method based on incremental stiffness matrices for the on-orbit growth of hundred-meter truss structures, discussing the multi-physics coupling of orbit, attitude, and flexible appendages under varying assembly paths. Additionally, Jiang et al. [14] leveraged a local translational coordinate frame to isolate large-scale orbital motion, effectively circumventing the loss of numerical precision inherent in traditional absolute coordinate methods during multi-scale coupling simulations. Although these works have achieved breakthroughs at the numerical simulation level, their modeling frameworks often lack sufficient consideration of practical manufacturing constraints when transitioning to ground-based experimental testing at extreme scaling ratios.

Regarding scaling theory and ground testing of space structures, major global space agencies [15,16] have invested substantial resources in the construction of various gravity-offloading testbeds. However, theoretical investigations into the physical laws of scaling equivalence remain limited. Zong et al. [17] derived complete similarity criteria for flexible space manipulators based on the classical Buckingham π theorem and optimized the mass and stiffness distribution of scaled models through structural sensitivity analysis. Zhang et al. [18] conducted preliminary explorations of local equivalence laws under partial geometric non-similarity for large membranes and flexible mechanisms. Qi et al. [19] proposed a scaled equivalent modeling framework merging geometrically exact theory with structural sensitivity analysis, aiming to overcome manufacturing bottlenecks and challenges in high-precision dynamic prediction for ultra-large space multibody systems through the optimization of similarity constants and local parameter adjustments. In fact, in fields such as aeroelasticity and hydrodynamics, “distortion scaling” theory has been widely employed to resolve engineering dilemmas where strict geometric similarity cannot be maintained [20,21]. It must be emphasized that large modular parabolic antennas are essentially complex space multibody systems composed of nonlinear hinges and thick-walled rods with high aspect ratios. Strict adherence to traditional “complete geometric similarity” for large-ratio scaling (e.g., 1/10 or smaller) would force the geometric dimensions of the already slender tubes into the submillimeter regime. This not only transcends the fabrication limits of aerospace carbon fiber reinforced polymers (CFRP) but also introduces critical manufacturing tolerances. The contradiction between theoretical similarity and practical manufacturing capabilities has led to significant fabrication bottlenecks for traditional complete equivalence theories in large-scale engineering practice.

To circumvent the fabrication bottlenecks imposed by strict geometric constraints, this paper investigates the dynamic equivalence between full-scale on-orbit assembled structures and ground-based scaled models, proposing a dynamic scaling method based on geometric distortion and material parameter compensation. This approach constructs a core dimensional space based on the three fundamental physical quantities: mass (M), length (L), and time (T) [22]. Through rigorous dimensional analysis, the geometric similarity constraints on the cross-sections of thick-walled rods are moderately relaxed. Simultaneously, low-modulus polymers are utilized to replace the CFRP material of the prototype to compensate for stiffness and mass errors induced by geometric distortion.

For numerical validation, single rods and two-bar mechanisms with spherical joints—which determine the overall stiffness of the truss—were selected as the primary research objects. The computational results demonstrate that the proposed strategy constrains the lower-order primary frequency errors of both the basic units and two-bar mechanisms to within 1%, under both free-floating and rail-constrained boundary conditions. This study effectively avoids the manufacturing challenges triggered by the large-scale reduction in thick-walled rods, providing a high-fidelity and manufacturable methodology for dynamic modeling and analysis of modular on-orbit assembled structures.

## 2. Abstraction of Research Objects and Theoretical Framework for Geometric-Material Distortion Similarity

The core of scaling for ultra-large space structures lies in establishing a precise dynamic equivalent relationship between the full-scale prototype and the small-scale equivalent model. For the basic physical units constituting these massive space structures—flexible rods with large aspect ratios and their modular mechanisms—their low-frequency dynamic responses, dominated by transverse bending, are the most critical factors influencing the stability of on-orbit assembly. This section first elucidates the dimension reduction and decoupling process of the research object, then rigorously derives the dynamic distortion scaling laws, and ultimately formulates compensation criteria for geometric dimensions and material properties.

### 2.1. Dimension Reduction and Decoupling from Ultra-Large Space Antenna Trusses to Basic Mechanical Primitives

Ultra-large space parabolic antennas possess immense physical volumes and are typically engineered by assembling standard hexagonal modules into arrays in orbit. Further observation of the local configurations reveals that each independent module is a polyhedral framework composed of slender truss members, which are usually connected by spatial nodes such as synchronous hinges and T-hinges [23]. The configuration of the robot-assembled module for the ultra-large parabolic antenna is illustrated in Figure 1. Confronted with such a flexible multibody system featuring numerous degrees of freedom and complex topological relationships, directly deriving and verifying the distortion similarity laws at the macroscopic system level would easily lead to a predicament of coupled nonlinear errors.

Therefore, it is necessary to perform structural dimension reduction and decoupling on the complex spatial trusses. In engineering practice, the kinematic accuracy of module folding and deployment is typically verified first by relying on perfectly similar rigid linkage mechanisms. Building upon this baseline, to ascertain the transmission laws of flexible distortion effects across nonlinear nodes, the research focus can be further narrowed to a flexible two-bar mechanism that contains a spatial spherical joint. The two-bar mechanism not only retains the fundamental flexible deformation characteristics of the rods but also introduces critical physical connection boundaries, serving as the core link to verify whether underlying distortion effects can achieve system-level equivalence. If all complex connections are stripped away, the single rod, as the most basic continuum and load-bearing carrier of the entire truss network, naturally emerges as the most fundamental physical unit for deriving the theory of cross-sectional geometric distortion and material parameter compensation.

Following the abstraction of the research objects, the subsequent step entails translating the assembly working conditions into mechanical boundary conditions. During on-orbit assembly, the basic single rod and linkage mechanisms operate in a microgravity environment; hence, their frequency extraction is primarily based on a completely unrestrained free boundary. In the ground validation phase, to counteract the influence of gravity on the flexible structures, the components are generally mounted on a rail support system. In theoretical equivalence, this configuration is simplified to a sliding rail boundary that allows exclusively uniaxial displacement. This setting primarily accounts for the necessity of displacement compensation in the extension direction during the module folding, deployment, or assembly docking processes. By releasing the axial constraint, the dynamic response of the modules during the assembly stroke can be simulated more realistically. Comparing the performance disparities between the free-floating state and this constrained state allows for a rigorous investigation into the dynamic robustness of the distorted model under practical constraint conditions.

### 2.2. Limitations of Classical Dimensional Analysis in Distortion Scaling

The prerequisite for investigating the free vibration characteristics of slender space rods is to first identify the core physical variables governing their dynamic behavior. These variables include the target natural frequency $\omega [T^{-1}]$, structural length L[L], characteristic cross-sectional dimension (e.g., cross-sectional diameter) D[L], material elastic modulus $E[ML^{-1}T^{-2}]$, and material density $\rho [ML^{-3}]$. The fundamental dimensions of each variable in the Mass–Length–Time (MLT) system are typically denoted in brackets. When the distortion variables are selected, the three fundamental variables L, E, and ρ cover all the fundamental dimensions (i.e., M, L, and T) needed to build the dimensionless π groups.

Based on the Buckingham π theorem, by establishing a dimensional matrix with these fundamental variables, the following two dimensionless π groups can be derived:

(1) $$\pi_{1}=\omega L\sqrt{\frac{\rho }{E}}$$

(2) $$\pi_{2}=\frac{D}{L}$$

According to the similarity principle, for the scaled model (subscript m) and the prototype (subscript p) to maintain complete dynamic similarity, their dimensionless numbers must be strictly equal. That is $\pi_{1m}=\pi_{1p}$ and $\pi_{2m}=\pi_{2p}$. Among them, $\pi_{2m}=\pi_{2p}$ corresponds to the complete geometric similarity criterion, which dictates that the length and cross-sectional dimensions must be scaled by the identical ratio (i.e., $\lambda_{l}=\lambda_{D}$). Under the premise of satisfying this geometric constraint, the classical frequency scaling mapping relationship can be directly derived from $\pi_{1m}=\pi_{1p}$:

(3) $$\lambda_{\omega }=(1/\lambda_{l})\sqrt{\lambda_{E}/\lambda_{\rho }}$$

However, when performing large-ratio scaling on space rods, strictly adhering to $\pi_{2m}=\pi_{2p}$ means that the cross-sectional dimensions of the model rods will shrink below the limits of current manufacturing processes, rendering them difficult to machine. To ensure machinability and practical feasibility, geometric distortion of the cross-section (i.e., $\lambda_{d}\neq \lambda_{l}$) is frequently introduced in engineering practice, which directly changes the slenderness ratio of the system and leads to $\pi_{2m}\neq \pi_{2p}$. At this stage, as the similarity equilibrium of $\pi_{2}$ is disrupted, the classical dimensional analysis method can hardly provide an explicit theoretical compensation mechanism; in other words, it cannot offset the dynamic deviations caused by geometric distortion by adjusting the physical material properties in $\pi_{1}$. Evidently, relying solely on the traditional similarity principles cannot satisfy the design requirements of distortion scaling. It is imperative to analyze the mechanical essence of the system and directly extract similarity indicators.

### 2.3. Extraction of Similarity Indicators Based on Partial Differential Equations

For flexible rods with large slenderness ratios, under the assumption of small elastic deformations, their transverse free vibration can be accurately described by the Euler–Bernoulli beam theory. The governing partial differential equation is expressed as:

(4) $$EI\frac{\partial^{4}y(x,t)}{\partial x^{4}}+\rho A\frac{\partial^{2}y(x,t)}{\partial t^{2}}=0$$

where y(x,t) is the transverse displacement field, x is the axial coordinate, t is time, A is the cross-sectional area, and I is the cross-sectional moment of inertia. Introducing independent scaling factors $\lambda_{k}=k_{m}/k_{p}$ for each physical quantity, the scaling relationships for space, time, displacement, and physical properties can be expressed as: $x_{m}=\lambda_{l}x_{p}$, $t_{m}=\lambda_{t}t_{p}$, $y_{m}=\lambda_{y}y_{p}$, $E_{m}=\lambda_{E}E_{p}$, $I_{m}=\lambda_{I}I_{p}$, $\rho_{m}=\lambda_{\rho }\rho_{p}$, and $A_{m}=\lambda_{A}A_{p}$. Substituting these scaled variables into the prototype’s differential equation and extracting the scaling factors as leading coefficients yields the model equation:

(5) $$\left(\frac{\lambda_{l}^{4}}{\lambda_{E}\lambda_{I}\lambda_{y}}\right)E_{m}I_{m}\frac{\partial^{4}y_{m}}{\partial x_{m}^{4}}+\left(\frac{\lambda_{t}^{2}}{\lambda_{\rho }\lambda_{A}\lambda_{y}}\right)\rho_{m}A_{m}\frac{\partial^{2}y_{m}}{\partial t_{m}^{2}}=0$$

As shown in Equation (5), the displacement scaling factor $\lambda_{y}$ can be canceled directly as a common factor, which proves that the natural frequencies of linear elastic systems are independent of vibration amplitude. Furthermore, boundary conditions are mathematically expressed as homogeneous constraints on the displacement field and its spatial derivatives; consequently, the scaling factor $\lambda_{y}$ also cancels out exactly in the boundary equations. For instance, in on-orbit assembly modules, the internal rods are connected via spatial spherical joints. These physical joints prevent relative translation between connected members while permitting relative rotation. In the theoretical model, such joints are explicitly represented by homogeneous constraints that enforce displacement continuity and zero bending moments at the connections. Since these physical constraints are governed by linear homogeneous equations, the $\lambda_{y}$ terms on both sides cancel out exactly. This demonstrates that the proposed dynamic similarity relationships are independent of specific external boundaries and physical joint configurations.

After the influence of the displacement factor $\lambda_{y}$ and boundary conditions has been eliminated, the remaining coefficients in Equation (5) must be kept equal. This ensures that the scaled model obeys the same governing differential equation as the prototype, and therefore preserves the same physical laws and mode shapes. Consequently, the governing equations for dynamic similarity are derived as follows:

(6) $$\frac{\lambda_{l}^{4}}{\lambda_{E}\lambda_{I}}=\frac{\lambda_{t}^{2}}{\lambda_{\rho }\lambda_{A}}$$

Since the natural frequency ω and the characteristic time t are reciprocals of each other (i.e., $\lambda_{\omega }=1/\lambda_{t}$), substituting this relationship and rearranging the terms yields the generalized frequency mapping equation:

(7) $$\lambda_{\omega }=\frac{1}{\lambda_{l}^{2}}\sqrt{\frac{\lambda_{E}}{\lambda_{\rho }}}\sqrt{\frac{\lambda_{I}}{\lambda_{A}}}$$

This equation indicates that the dynamic scaling of the structure is strongly correlated with three distinct components: macroscopic geometric scaling ($1/\lambda_{l}^{2}$), material property scaling ($\sqrt{\lambda_{E}/\lambda_{\rho }}$), and microscopic cross-sectional shape scaling ($\sqrt{\lambda_{I}/\lambda_{A}}$).

### 2.4. Construction of Geometric-Material Compensation Criteria Based on Equivalent Continuum Benchmark

In authentic aerospace engineering practice, ultra-large-span trusses predominantly utilize carbon fiber thin-walled or thick-walled hollow tubular components. However, directly introducing hollow cross-sections in theoretical studies of system-level dynamic scaling laws introduces additional complications. For tubular components with a certain thickness, the actual wall thickness t cannot simply be eliminated during theoretical derivations. It significantly alters the bending characteristics of the cross-section, thereby directly affecting the vibration mode shapes of the entire rod during bending vibration. Furthermore, finite element models incorporating actual wall thicknesses are highly susceptible to inducing local shell vibrations or wall buckling during computation, which obscures the global dynamic response of genuine interest. Therefore, to purely investigate the inherent laws of geometric distortion and material compensation while excluding the interference of the complex wall thickness variable, the preliminary theoretical derivations and numerical simulation validations in this paper are based on solid circular continua. The equivalent verification for hollow structures will be specifically discussed in subsequent sections.

For a solid circular cross-section basic unit defined by an equivalent characteristic diameter D—a dimension that inherently encapsulates the geometric influence of the actual hollow tube’s wall thickness—the cross-sectional area A and cross-sectional moment of inertia I are, respectively:

(8) $$A=\frac{\pi D^{2}}{4}$$

(9) $$I=\frac{\pi D^{4}}{64}$$

The cross-sectional radius of gyration $r_{g}$ extracted from this exhibits a strict linear proportional relationship with the cross-sectional diameter D:

(10) $$r_{g}=\sqrt{\frac{I}{A}}=\frac{D}{4}$$

When establishing the mapping relationship between the full-scale prototype and the distorted scaled model, the microscopic cross-sectional shape scaling factor $\sqrt{\lambda_{I}/\lambda_{A}}$ can be directly simplified to the scaling ratio of the cross-sectional diameter (i.e., the cross-sectional distortion ratio $\lambda_{D}$):

(11) $$\sqrt{\frac{\lambda_{I}}{\lambda_{A}}}=\frac{D_{m}/4}{D_{p}/4}=\lambda_{D}$$

Substituting this cross-sectional mapping relationship into the generalized dynamic equivalent equation yields the parametric distortion scaling law that is applicable to the solid continuum benchmark.

(12) $$\lambda_{\omega }=\frac{\lambda_{D}}{\lambda_{l}^{2}}\sqrt{\frac{\lambda_{E}}{\lambda_{\rho }}}$$

For the ground testing of ultra-large space antennas during on-orbit assembly, the primary objective is to ensure a 1:1 absolute equivalence of the critical lower-order primary frequencies between the prototype and the scaled model, thereby enabling direct extrapolation of ground tests to on-orbit dynamic characteristics. By setting the frequency scaling factor $\lambda_{\omega }=1$, the explicit equivalent compensation criterion for geometric distortion and material substitution can be formally derived:

(13) $$\sqrt{\frac{\lambda_{E}}{\lambda_{\rho }}}=\frac{\lambda_{l}^{2}}{\lambda_{D}}\;\Rightarrow \;{\left(\frac{E}{\rho }\right)}_{m}={\left(\frac{E}{\rho }\right)}_{p}\cdot \frac{\lambda_{l}^{4}}{\lambda_{D}^{2}}$$

This compensation criterion reveals the cross-dimensional self-consistent mechanism in structural dynamics at a fundamental mathematical and physical level. The equation explicitly indicates that, to circumvent the micro-dimensional machining impasse brought about by the significant scaling down of slender structures (i.e., $\lambda_{l}\ll 1$), engineering physical modeling permits the deliberate relaxation of geometric constraints to substantially amplify the characteristic diameter of the rods (i.e., $\lambda_{D}\gg \lambda_{l}$). The nonlinear deviations in the stiffness and mass matrices caused by this violation of the complete similarity criterion must be effectively offset by the precise selection of model materials with extremely low specific stiffness $(E/\rho {)}_{m}$. Among commonly used engineering materials, polymers such as polytetrafluoroethylene (PTFE) and polyurethane (PU) not only possess extremely low linear elastic moduli but also feature stable density distributions and excellent precision machinability. In fact, these two flexible polymers are selected as physical surrogate baselines primarily as representative candidates to demonstrate the mathematical and physical feasibility of the proposed parameter compensation theory; other polymers with appropriate specific stiffness are equally applicable. The physical parameters of PTFE and PU used herein are selected from the typical parameter ranges provided in Reference [24]. This compensation strategy provides rigorous theoretical support for the subsequent high-fidelity numerical validation using solid beam elements, and significantly expands the material design space for low-cost ground physical testing of ultra-large-scale space structures.

## 3. Numerical Simulation Analysis of the Geometric-Material Distortion Similarity Theory

### 3.1. Numerical Simulation Models and Boundary Conditions

Adhering to the dimension reduction and decoupling strategy outlined in Section 2.1, this section validates the effectiveness of the geometric-material distortion similarity theory proposed in Section 2 by using numerical simulation results under multiple operating conditions. The proposed method is initially validated using a single rod. Subsequently, a two-bar mechanism is employed to demonstrate the method’s effectiveness within a multibody kinematic system. Finally, constrained boundary conditions are introduced to investigate the adaptability of the proposed strategy to external constraints. This progressive validation approach enables a comprehensive analysis of the actual performance of the distortion equivalent strategy in multibody structures, providing a theoretical foundation for subsequent research on deployable modular assemblies.

In constructing the finite element (FE) models, to accurately characterize the cross-sectional mechanical effects following distortion, the structural rods are uniformly modeled using two-node linear spatial beam elements (B31) within a commercial FE software environment. This element effectively accounts for the bending deformation of the rods and the cross-sectional moment of inertia, closely aligning with the authentic mechanical characteristics of the equivalent continuum. Addressing the critical kinematic joints inherent in the two-bar mechanism, the simulation models accurately replicate the spatial spherical joints at the assembly nodes using connector elements. The incorporation of these kinematic joints serves to rigorously verify whether the distortion compensation characteristics of the rods remain effective upon the introduction of complex nonlinear physical nodes, thereby maintaining a high degree of consistency in the system-level frequency mapping.

**Figure 2:**
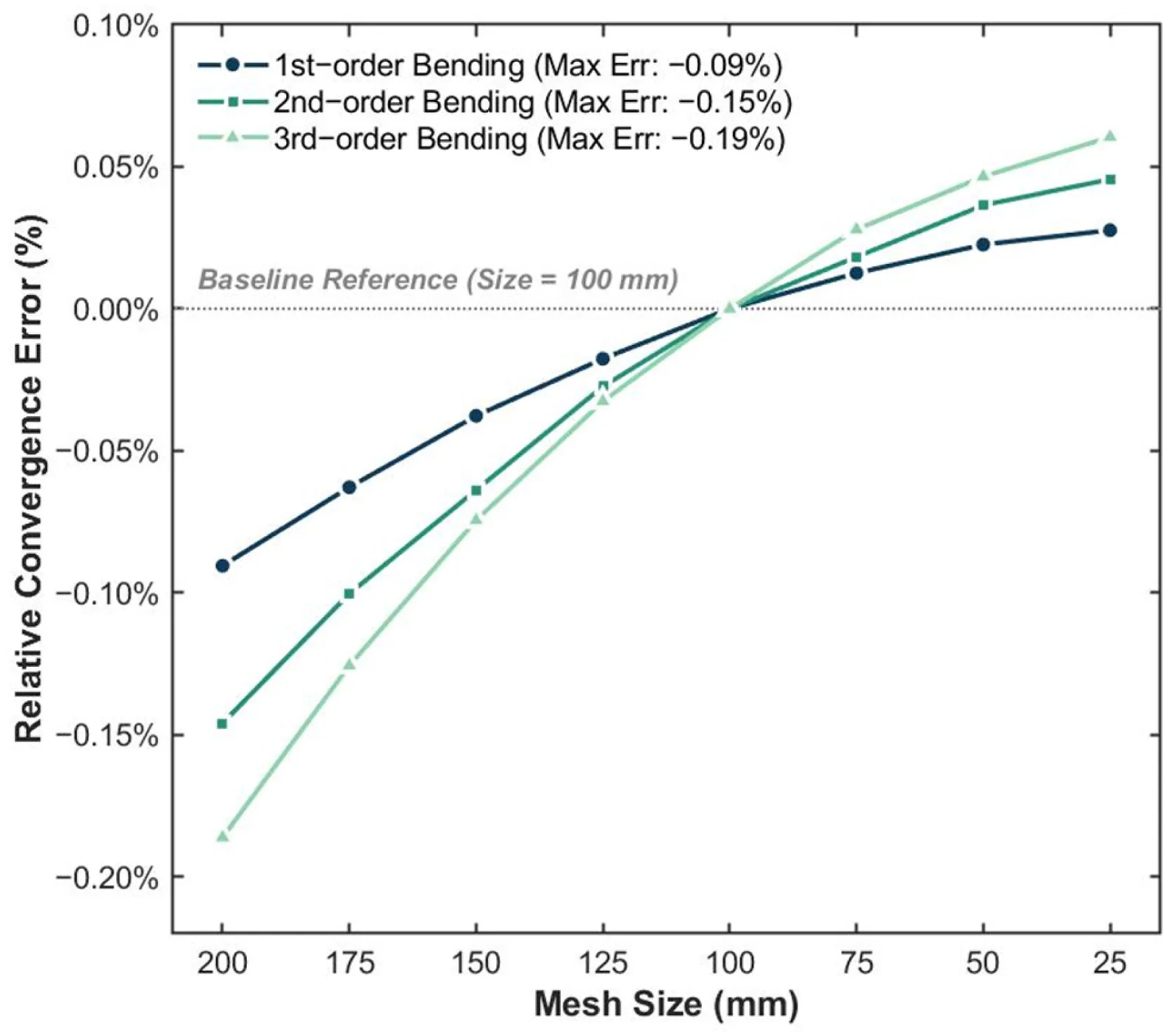
Mesh convergence analysis of the first three bending frequencies of the prototype single-rod.

The setup of boundary conditions is strictly differentiated according to the specific physical validation scenarios. For the models simulating the on-orbit phase, no constraints are applied; completely unconstrained boundary conditions are adopted to extract the elastic modes of the structure within a microgravity floating environment. In contrast, to emulate the actual conditions of air-bearing offloading and docking compensation during ground tests, the constrained simulation group incorporates a sliding directional boundary at the ends of the components. This boundary rigidly constrains partial translations and all spatial rotations of the nodes, releasing only the extensional degree of freedom along the axial direction of the rod. To eliminate the interference of mesh size on dynamic calculation accuracy, the mesh size is progressively refined from 200 mm to 25 mm, and the first three bending frequencies of the structure are extracted and compared. Using the results obtained with a 100 mm mesh size as the baseline reference, Figure 2 illustrates the evolution trend of the relative errors for these frequencies.

As shown in Figure 2, relative to the 100 mm baseline, even when the mesh is further refined to 25 mm, the relative deviation of the frequencies remains less than 0.1%. This indicates that a mesh size of 100 mm sufficiently satisfies the convergence requirements. Balancing computational efficiency and accuracy, the 100 mm mesh size is selected as the unified standard for all subsequent simulations.

### 3.2. Validation of Dynamic Equivalence and Parameter Compensation for the Single-Rod Component

**Figure 3:**
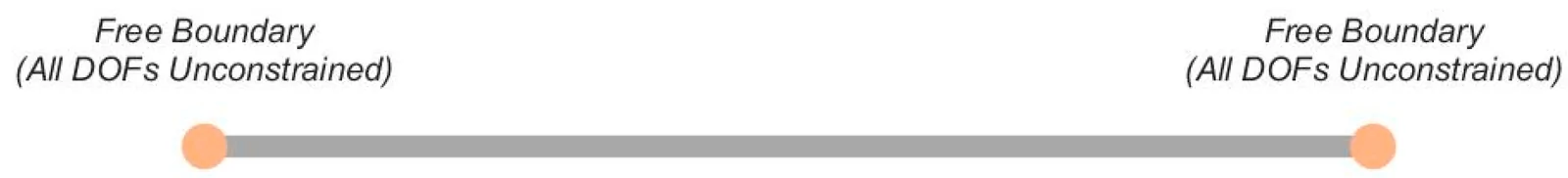
Schematic diagram of the boundary conditions for the unconstrained single rod.

This set of numerical simulations focuses on the single-rod unit; the detailed structural schematic is illustrated in Figure 3, and the geometric dimensions and material parameters are presented in Table 1. The full-scale prototype (Case 1-PRO) utilizes a CFRP solid rod, with a nominal length L of 10,000 mm and an equivalent cross-sectional diameter D of 50 mm. To achieve dynamic validation at the scaled level, the lengths of the scaled models were uniformly set to 2000 mm, establishing a specific length scaling ratio (i.e., $\lambda_{l}=1/5$). Among them, Case 1-SIM serves as the traditional complete geometric similarity baseline group commonly used in engineering; its cross-sectional diameter was strictly scaled down proportionally to 10 mm, while the material physical properties (elastic modulus and density) were kept identical to those of the prototype. However, this traditional scaling approach presents a critical limitation in dynamic equivalence: because no material substitution was performed, its natural frequencies at each order are drastically amplified according to the corresponding similarity law. Moreover, as previously discussed, proportional scaling results in an extremely slender component, which is highly prone to inducing manufacturing difficulties and structural instability. The core validation groups in this section (Case 1-PTFE and Case 1-PU) applied the cross-sectional distortion and material compensation method. While maintaining the scaled length of 2000 mm constant, the solid cross-sectional diameters were disproportionately enlarged to 40 mm and 50 mm, respectively. In accordance with the geometric-material parameter compensation law derived in Section 2, polytetrafluoroethylene (PTFE) and polyurethane (PU) were matched as substitute materials for Case 1-PTFE and Case 1-PU, respectively.

The natural frequencies of each model in the completely unconstrained state were calculated using the built-in eigenvalue solver in Abaqus 2020. The extracted results for the first three primary bending frequencies and the statistical relative errors are summarized in Table 2.

**Figure 4:**
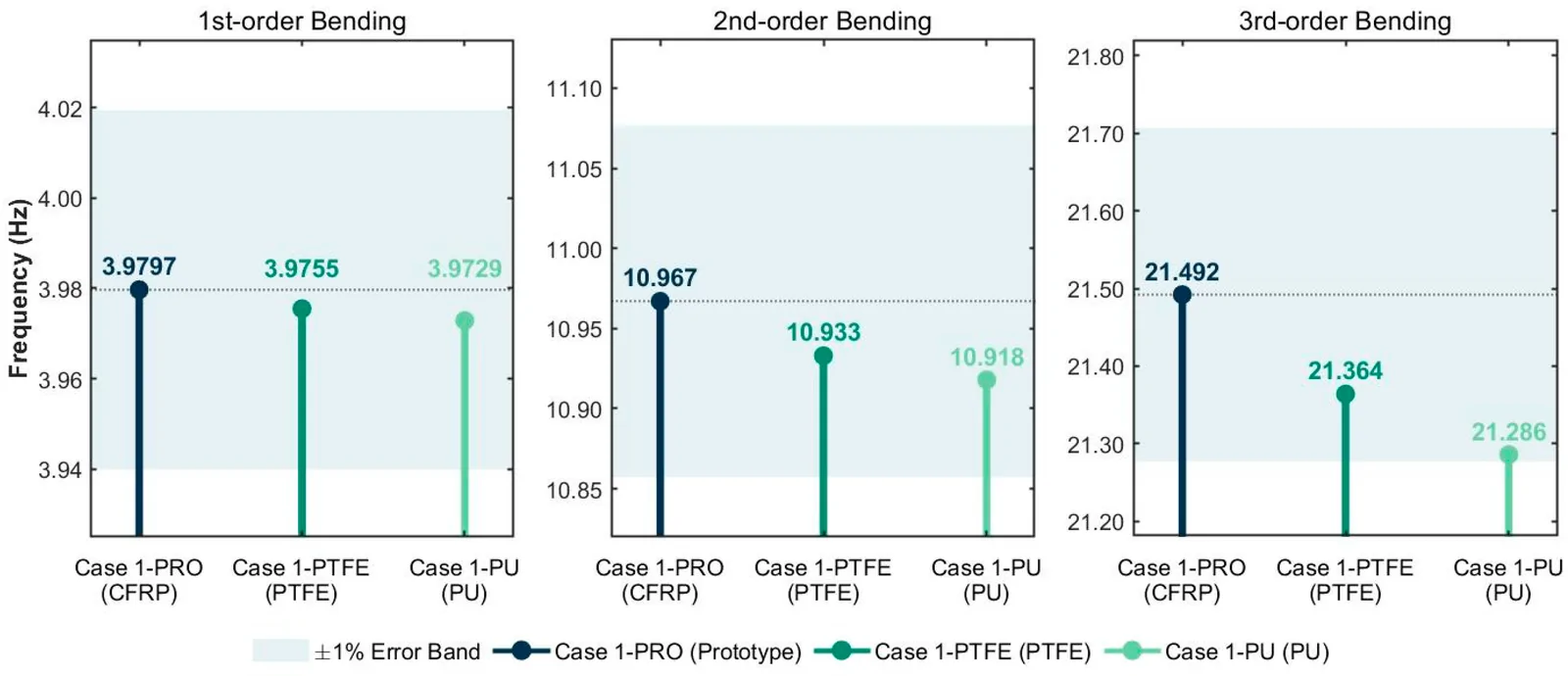
Comparative analysis of the first three elastic bending frequencies between the prototype and the distorted models within a ±1% error band.

The simulation data indicate that the deviations from engineering targets for the first three primary frequencies of the Case 1-SIM model, which adhered to the traditional complete similarity criterion, are all approximately +400%. This not only leads to a severe distortion of dynamic characteristics but also demonstrates the limitations of traditional similarity theory when handling such cross-scale equivalent models. Figure 4 illustrates the relative positional relationship between the frequencies of Case 1-PTFE and Case 1-PU, which applied the distortion method, and the ±1% error bands.

As shown in Figure 4, the frequency prediction errors for both the PTFE distorted model (Case 1-PTFE) and the PU distorted model (Case 1-PU) are within 1%, with a maximum relative error of −0.958%. Notably, as the modal order increases, the error exhibits a slight increasing trend. This occurs because, during the distortion design, the significant amplification of the cross-sectional diameter leads to a drastic decrease in the slenderness ratio of the rods. When extracting higher-order modes, this “moderately thick beam” characteristic makes the Timoshenko effect—induced by shear deformation and cross-sectional rotary inertia—more pronounced than in the prototype, thus introducing a minor deviation between the theoretical mapping and the finite element results. Nevertheless, an equivalent error constrained within 1% fully satisfies the stringent dynamic similarity accuracy requirements for ground testing of ultra-large space structures.

**Figure 5:**
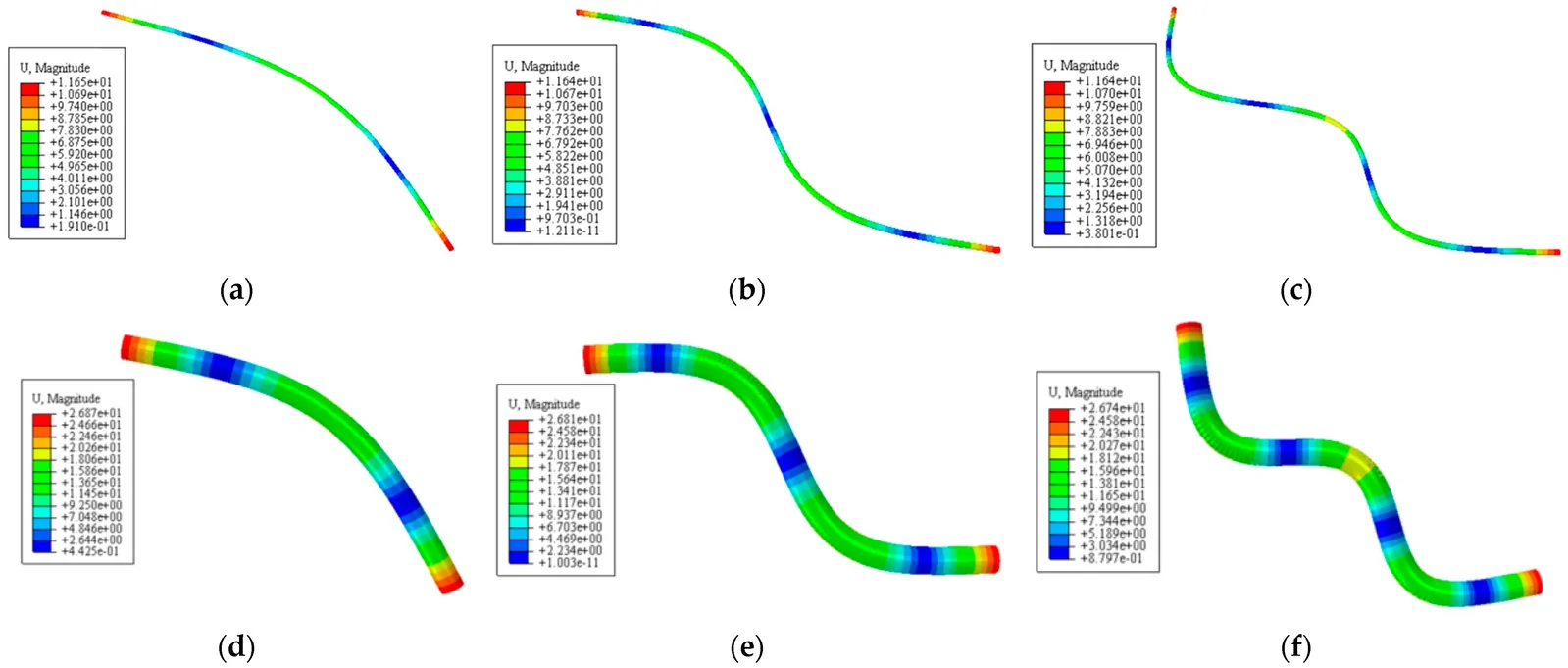
Comparison of the vibration mode shapes for Case 1-PRO and Case 1-PTFE. (**a**) 1st mode shape of Case 1-PRO; (**b**) 2nd mode shape of Case 1-PRO; (**c**) 3rd mode shape of Case 1-PRO; (**d**) 1st mode shape of Case 1-PTFE; (**e**) 2nd mode shape of Case 1-PTFE; (**f**) 3rd mode shape of Case 1-PTFE.

The high-precision mapping of frequencies solely validates the effectiveness of the proposed method at the numerical level; to demonstrate system-level dynamic similarity, it is necessary to further confirm the fidelity of the structural mode shapes. Given that the eigenvectors of the completely geometrically similar model (Case 1-SIM) are mathematically equivalent to those of the prototype, and considering that Case 1-PU follows the same physical compensation mechanism as Case 1-PTFE, only the comparison of mode shapes between the CFRP prototype (Case 1-PRO) and the PTFE distorted model (Case 1-PTFE) is presented here to avoid redundancy. The comparison of their first three mode shapes is shown in Figure 5.

**Figure 6:**
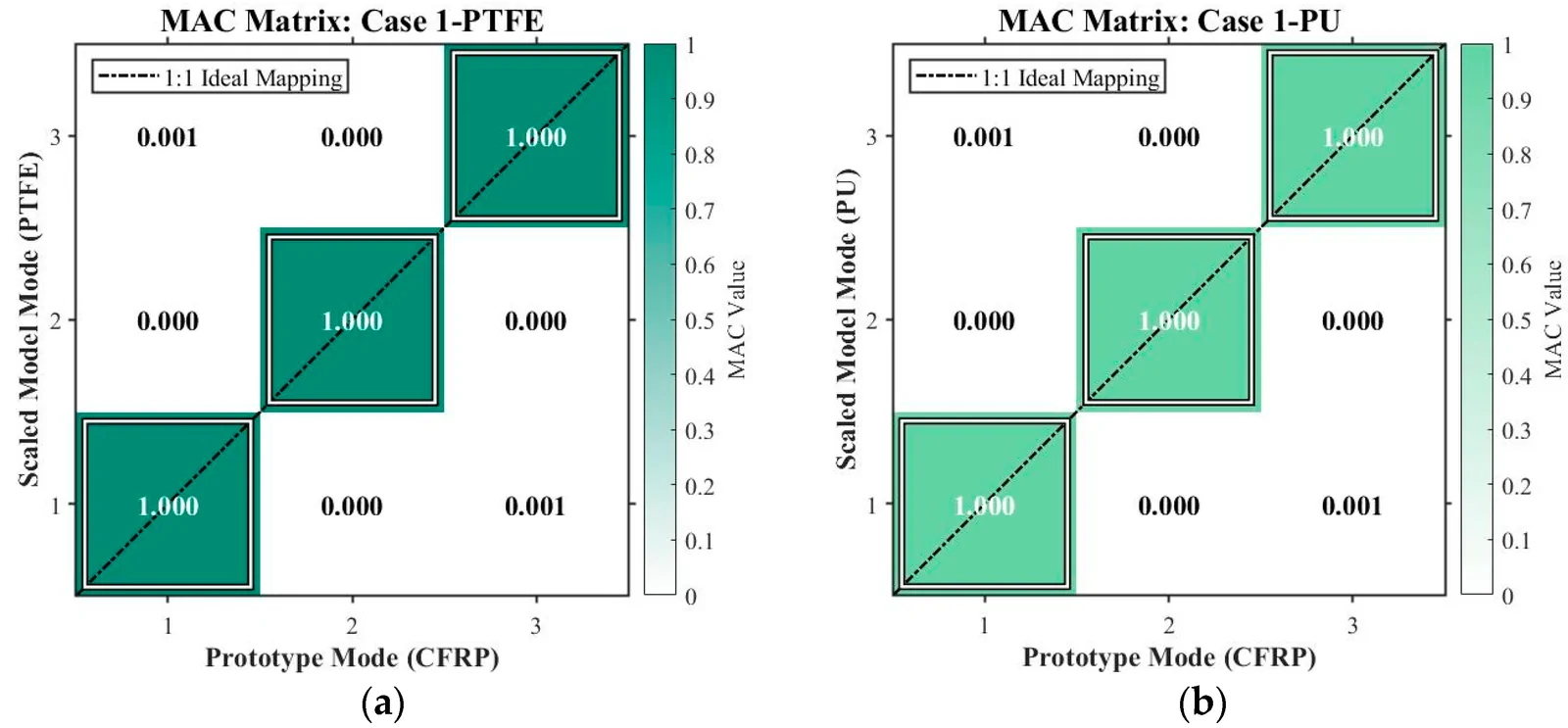
Modal Assurance Criterion (MAC) matrices between the CFRP prototype and the scaled models. (**a**) MAC matrix for Case 1-PTFE; (**b**) MAC matrix for Case 1-PU.

Furthermore, to quantitatively examine whether mode transitions occur during the parameter distortion process, the Modal Assurance Criterion (MAC) is introduced for a comprehensive evaluation. The MAC matrices between the CFRP prototype and the two distorted single-rod models are shown in Figure 6. The results indicate that the main diagonal elements of the MAC matrices for the first three bending modes of Case 1-PTFE and Case 1-PU all reach 1, exhibiting a prominent ideal 1:1 mapping relationship, while the off-diagonal elements all approach zero. These quantitative data fully corroborate that, even after the cross-sectional proportional constraints are broken, the distorted models perfectly retain the spatial orthogonality and mode shape contours of the prototype without any modal interchange. This finding effectively validates the applicability of the distortion equivalent theory to single-rod members.

**Figure 7:**
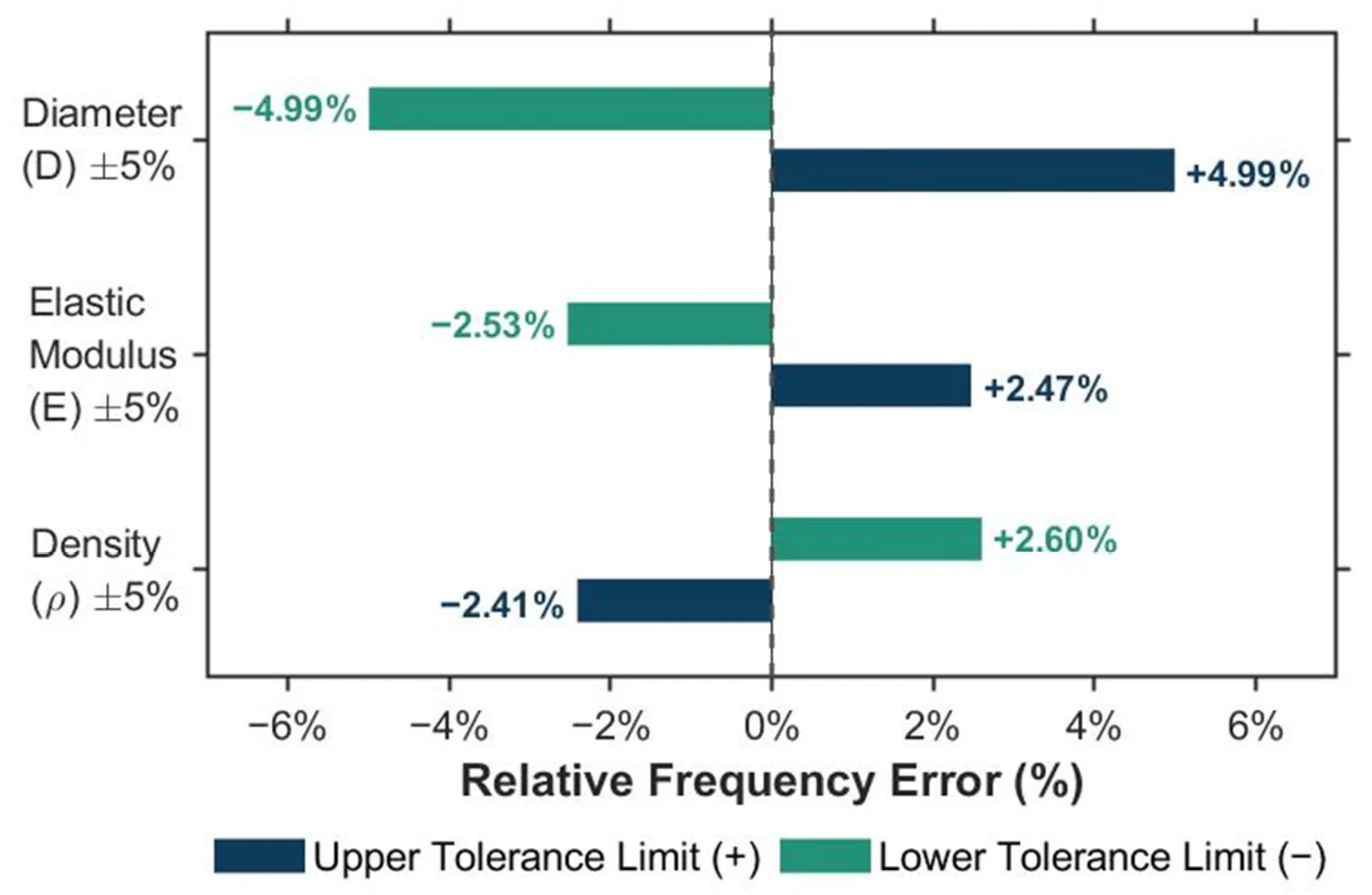
Sensitivity analysis of the first bending frequency under parameter uncertainties.

Moreover, in a room-temperature ground testing environment, the viscoelastic hysteresis effects of polymers induced by low-frequency vibration are relatively limited, yet minor fluctuations in ambient temperature can still cause the dynamic modulus and density of PTFE or PU to deviate from their nominal values. Therefore, the equivalent single-rod model (Case 1-PTFE) is selected to conduct the sensitivity and robustness analysis. The material parameter uncertainties caused by temperature fluctuations and slight viscoelasticity, alongside manufacturing tolerances, are uniformly formulated as realistic physical perturbations of ±5% in Young’s modulus (E), density (ρ), and rod diameter (D). The relative errors of the first-order bending natural frequency of the system are extracted and calculated, with the results illustrated in Figure 7. As can be observed, across the entire ±5% perturbation interval, the frequency deviations induced by all parameters are strictly enveloped within 5% (with a maximum relative error of ±4.99%). The frequency response exhibits a benign linear mapping characteristic, with no nonlinear error amplification occurring. This compellingly demonstrates that the proposed distortion equivalence method possesses exceptional fault tolerance and robustness against conventional manufacturing tolerances and material variations. This also implies that even when accounting for the inherent viscoelasticity and temperature dependence of the polymers, the frequency deviations induced within the realistic physical perturbation range remain entirely controllable. Furthermore, in the engineering design of the scaled model, the outer diameter dimension of the rods is not a strictly fixed rigid constraint. Before conducting actual physical tests, the machined outer diameter of the rods can be appropriately relaxed or fine-tuned based on the measured dynamic material modulus at the current ambient temperature. Such flexible geometric compensation can effectively offset the stiffness deviations caused by material property fluctuations, thereby further ensuring high fidelity in the dynamic predictions of ground tests.

### 3.3. Validation of Dynamic Equivalence and Assembly Transmissibility in Multibody Systems

This section extends the research object to a multibody assembly system containing spatial spherical joints. The introduction of spherical joints causes local stiffness discontinuities and endows the system with additional degrees of freedom. Therefore, this section targets the two-bar spatial mechanism as the core research focus, aiming to demonstrate the applicability of the proposed distortion similarity theory in multibody systems characterized by complex physical connections.

**Figure 8:**
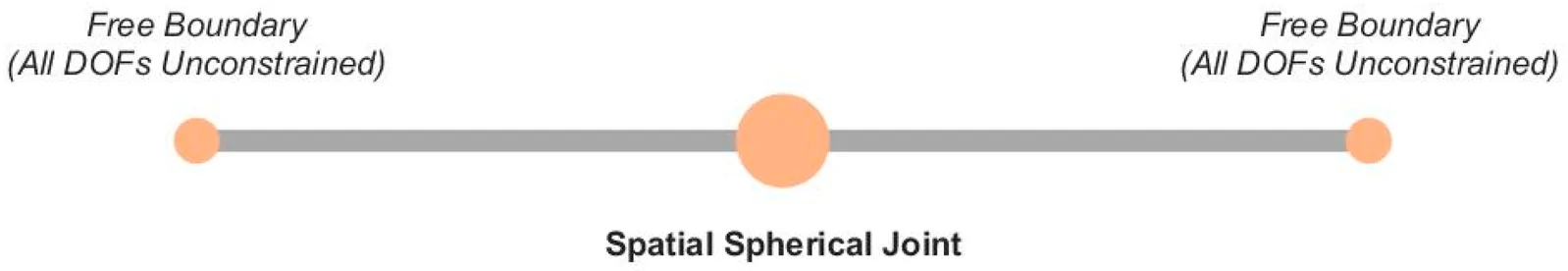
Schematic diagram of the boundary conditions for the unconstrained multibody system.

The rod parameters of the two-bar mechanism in this group were strictly inherited from the single-rod example in Section 3.2. Among them, the full-scale prototype system for the on-orbit operating condition (Case 2-PRO) was articulated from two CFRP rods (parameters identical to Case 1-PRO) via an ideal spatial spherical joint, with its initial configuration in a fully deployed straight-line state at 180°. The structural schematic is shown in Figure 8. As a theoretical control, the traditional similarity model (Case 2-SIM) was assembled using two strictly proportionally scaled CFRP slender rods (parameters identical to Case 1-SIM). Similarly, the core distortion validation groups in this section (Case 2-PTFE and Case 2-PU) were assembled using two non-proportionally enlarged PTFE rods (parameters identical to Case 1-PTFE) and PU rods (parameters identical to Case 1-PU), respectively. To strictly adhere to the control-variable principle, the models in this section retain the settings of Section 3.2 with unconstrained boundaries (i.e., free-free conditions), aiming to eliminate the interference of external constraints and solely verify the transferability of the theory across spherical joints.

The aforementioned multibody systems were solved using finite element analysis, and the obtained primary frequency data for each system are presented in Table 3. Given that the system initially assumes a 180° straight-line configuration, it possesses complete axisymmetry in three-dimensional space and lacks predefined in-plane or out-of-plane physical boundaries. Consequently, these three extracted frequencies physically correspond to the first-order, second-order (manifesting as a hinge-dominated V-shaped folding), and third-order global spatial bending of the multibody system, respectively.

**Figure 9:**
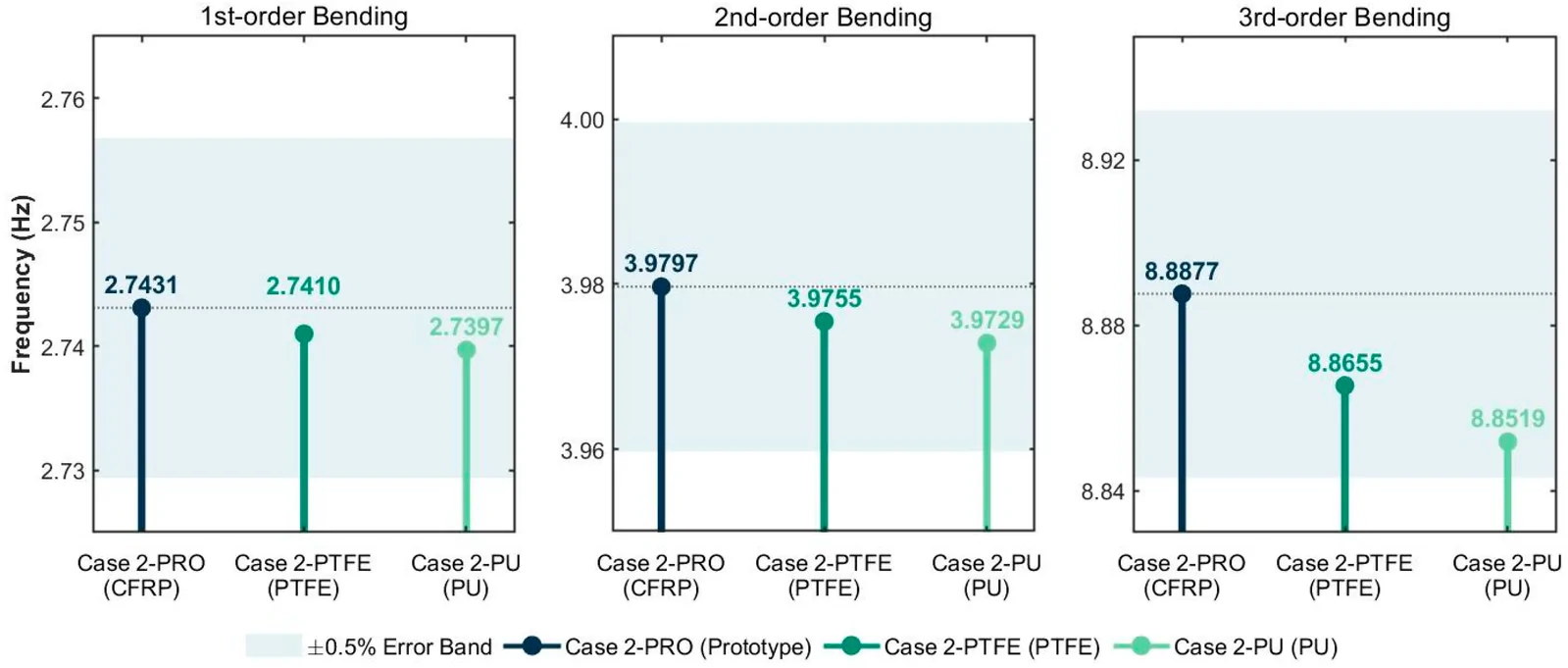
Comparative analysis of the first three system-level bending frequencies between the prototype and the distorted two-bar models, within a ±0.5% error margin.

A quantitative analysis of the data in Table 3 reveals that, as in Case 1-SIM, the deviations in the first three primary frequencies of the Case 2-SIM model, which adheres to the traditional geometric similarity criterion, are all approximately +400%. This once again corroborates the limitations of applying traditional geometric similarity methods to the dynamic scaling prediction of large-span space structures. The prediction errors for the first three system bending primary frequencies of both the PTFE distorted group (Case 2-PTFE) and the PU distorted group (Case 2-PU) are constrained within 0.5%, with a maximum error of −0.403%. Figure 9 illustrates the relative positional relationship between the frequencies of Case 2-PTFE and Case 2-PU and the ±0.5% error bands.

As depicted in the figure, the PTFE distorted model (Case 2-PTFE) and the PU distorted model (Case 2-PU) accurately reproduce the frequency response of the full-scale prototype system. The errors in the first three system primary frequencies for both distorted models remain within 0.5%, with a maximum error of −0.403%. This demonstrates that the geometric-material parameter substitution implemented on single components does not experience error amplification or accumulation during the global matrix assembly via spatial joints, thereby demonstrating the highly robust assembly transmissibility of the proposed method.

**Figure 10:**
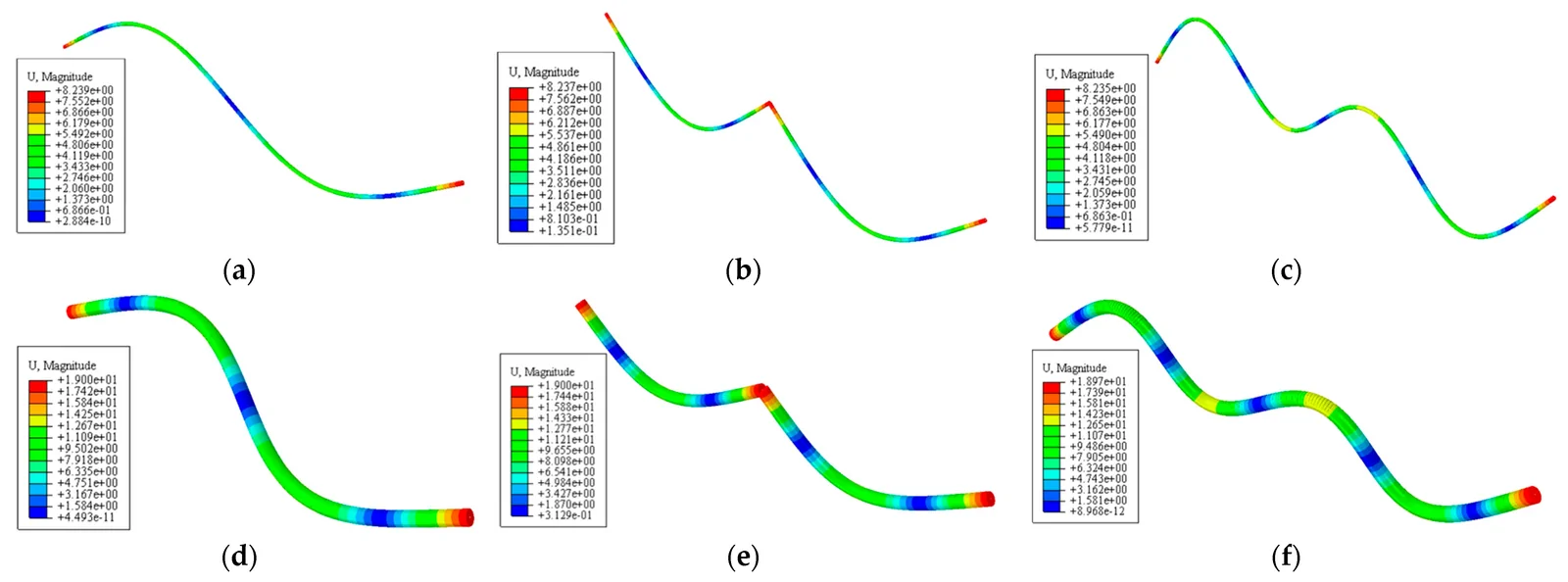
Comparison of the vibration mode shapes for Case 2-PRO and Case 2-PTFE: (**a**) first system bending mode of Case 2-PRO; (**b**) second system bending mode of Case 2-PRO; (**c**) third system bending mode of Case 2-PRO; (**d**) first system bending mode of Case 2-PTFE; (**e**) second system bending mode of Case 2-PTFE; (**f**) third system bending mode of Case 2-PTFE.

**Figure 11:**
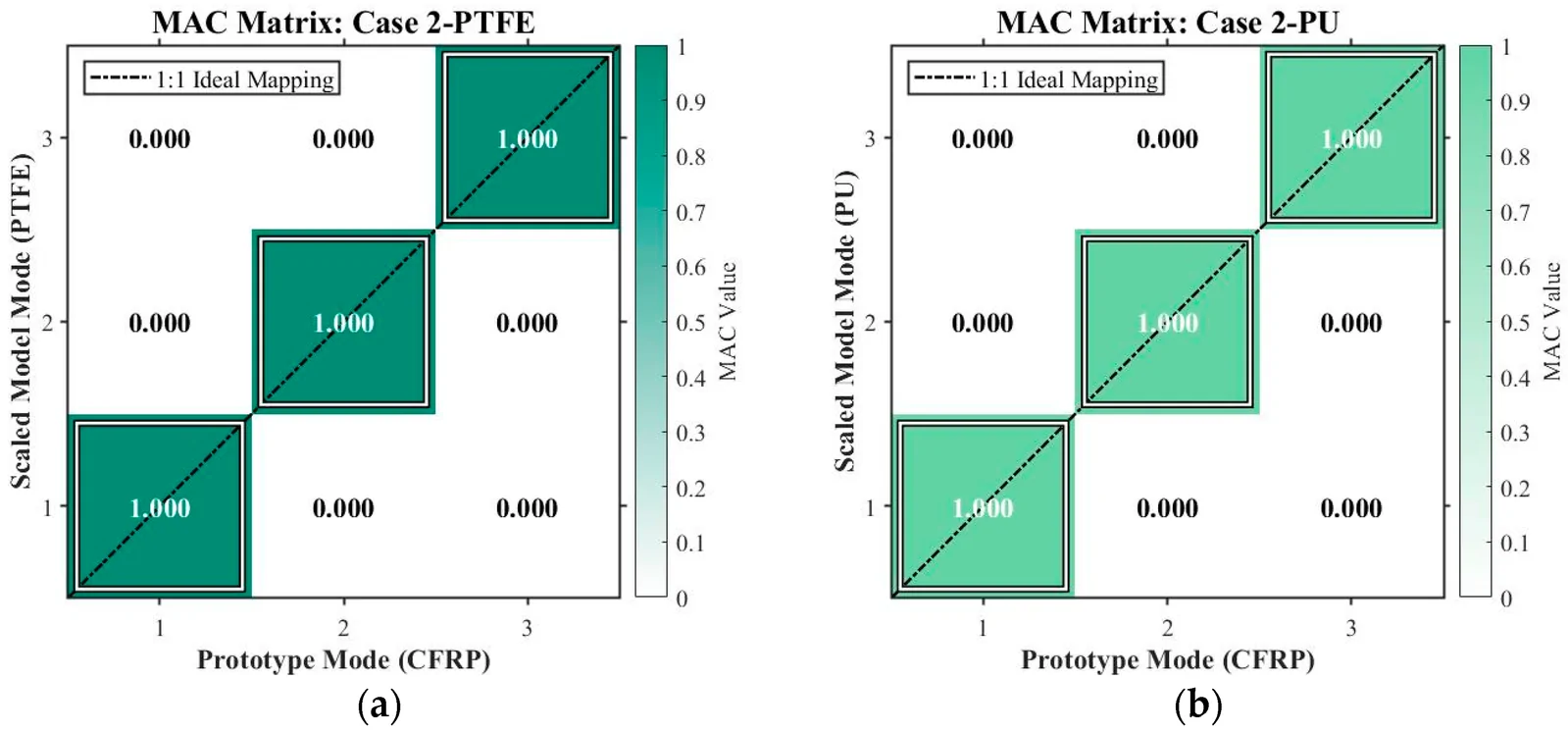
Modal Assurance Criterion (MAC) matrices between the CFRP prototype and the scaled models. (**a**) MAC matrix for Case 2-PTFE; (**b**) MAC matrix for Case 2-PU.

As in Section 3.2, Figure 10 displays the mode shape comparison between the prototype (Case 2-PRO) and the PTFE distorted group (Case 2-PTFE). As observed from the figure, the waveform profiles and node distributions of the two experimental groups remain highly consistent, and the relative rotation trends at the spherical joints perfectly align with the deformation characteristics of the rods. Figure 11 further presents the MAC matrices of the system mode shapes. The results show that, when compared with the prototype, the main diagonal values of the MAC matrices for the first three system bending modes of the two distorted two-bar models each reach 1.000, and the off-diagonal elements are all zero. This indicates that the theoretical errors induced by cross-sectional distortion and material compensation do not accumulate with the integration of multibody topologies; the theory successfully achieves an application leap from basic units to complex assemblies.

### 3.4. Dynamic Equivalence and Accuracy Validation of Constrained Multibody Systems

To verify the applicability of the proposed distortion similarity theory in actual engineering applications, this section introduces constrained boundary conditions based on the two-bar mechanism presented in Section 3.3 to re-evaluate the dynamic response of the system.

**Figure 12:**
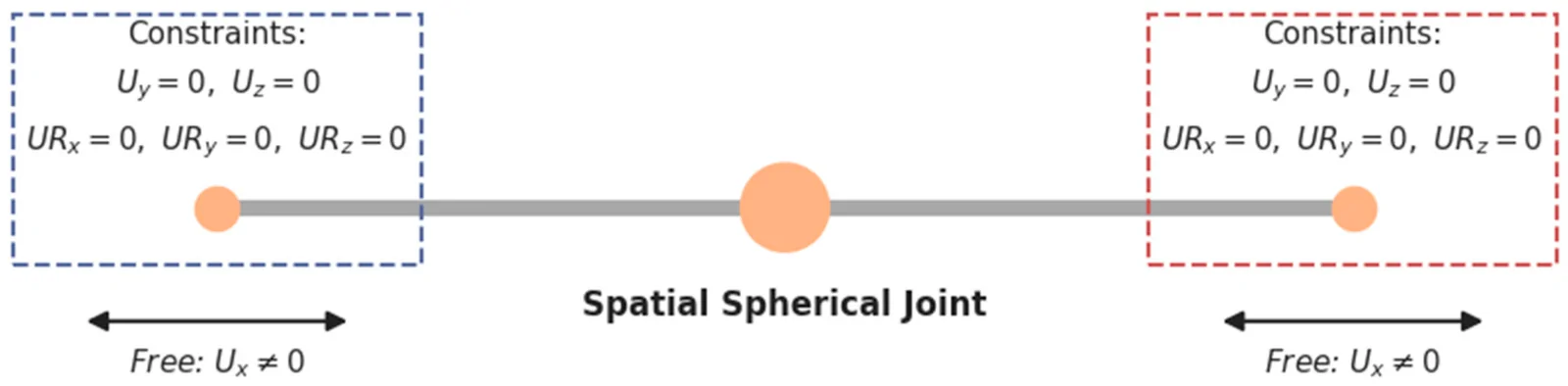
Schematic diagram of the boundary conditions for the constrained multibody system.

The system dimensions, rod connection relationships, and material parameters of the validation models in this group were kept entirely consistent with those in Section 3.3. However, constrained boundaries were imposed at the bilateral endpoints of the system: five degrees of freedom at the ends were constrained, encompassing translations in two directions and rotations in three directions, while only a single translational degree of freedom along the axial direction of the rods was retained at each end. A schematic diagram of these constraints is illustrated in Figure 12. This boundary condition effectively simulates the uniaxial frictionless sliding rail constraint scenarios commonly employed in spacecraft ground deployment tests. The alteration of boundary conditions inherently reconstructs the global stiffness matrix of the system, serving as a critical procedure for examining the boundary independence of the proposed method.

Under the aforementioned sliding rail constraints, the first three primary bending frequencies for each system were obtained using finite element analysis, and the results are summarized in Table 4.

**Figure 13:**
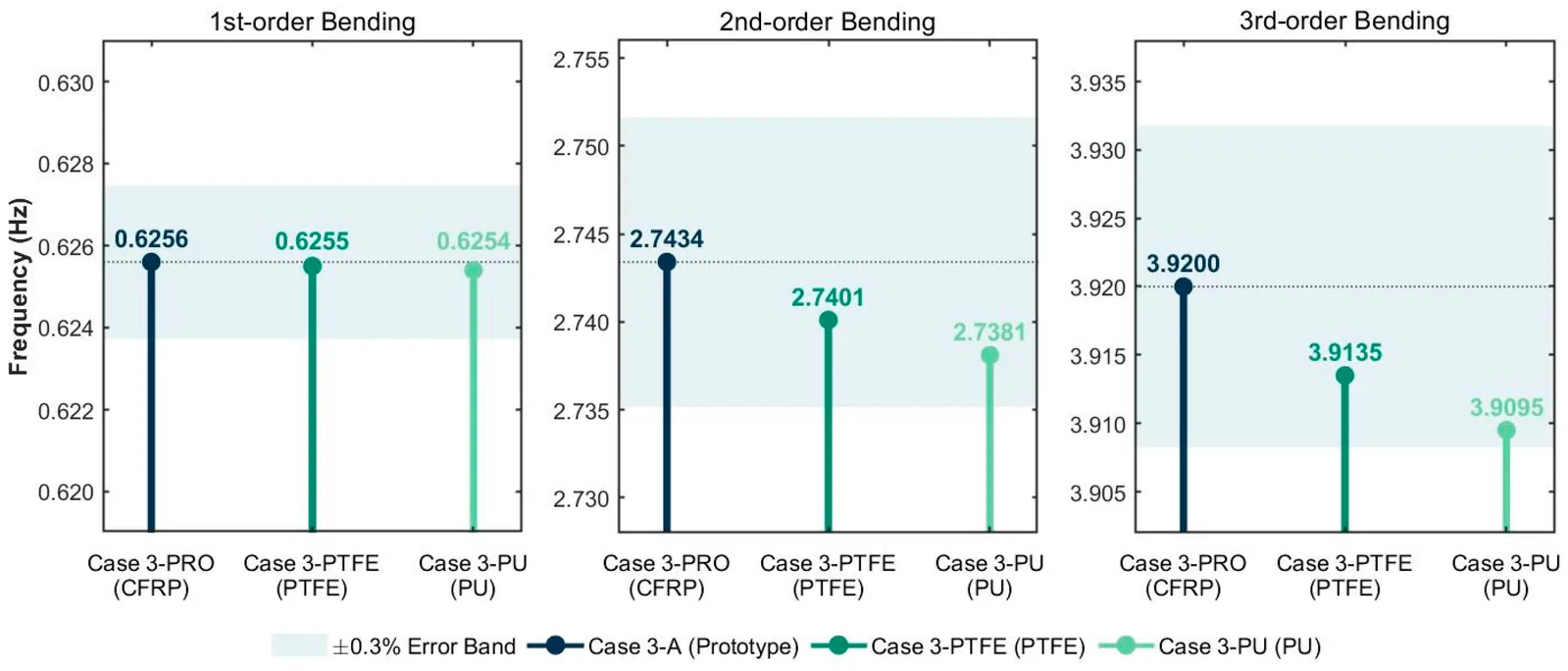
Comparative analysis of the first three system-level bending frequencies between the prototype and distorted two-bar models under constrained boundary conditions within a ±0.3% error band.

As illustrated in the table, the cross-sectional distortion and material compensation strategy maintain extremely high equivalent accuracy even after severe alterations to the boundary conditions. The prediction errors for the first three primary bending frequencies of both the PTFE distorted group (Case 3-PTFE) and the PU distorted group (Case 3-PU) are constrained within 0.3%, with a maximum error of −0.268%. Figure 13 illustrates the relative positional relationship between the frequencies of the distorted models under the constrained state and the ±0.3% error bands.

**Figure 14:**
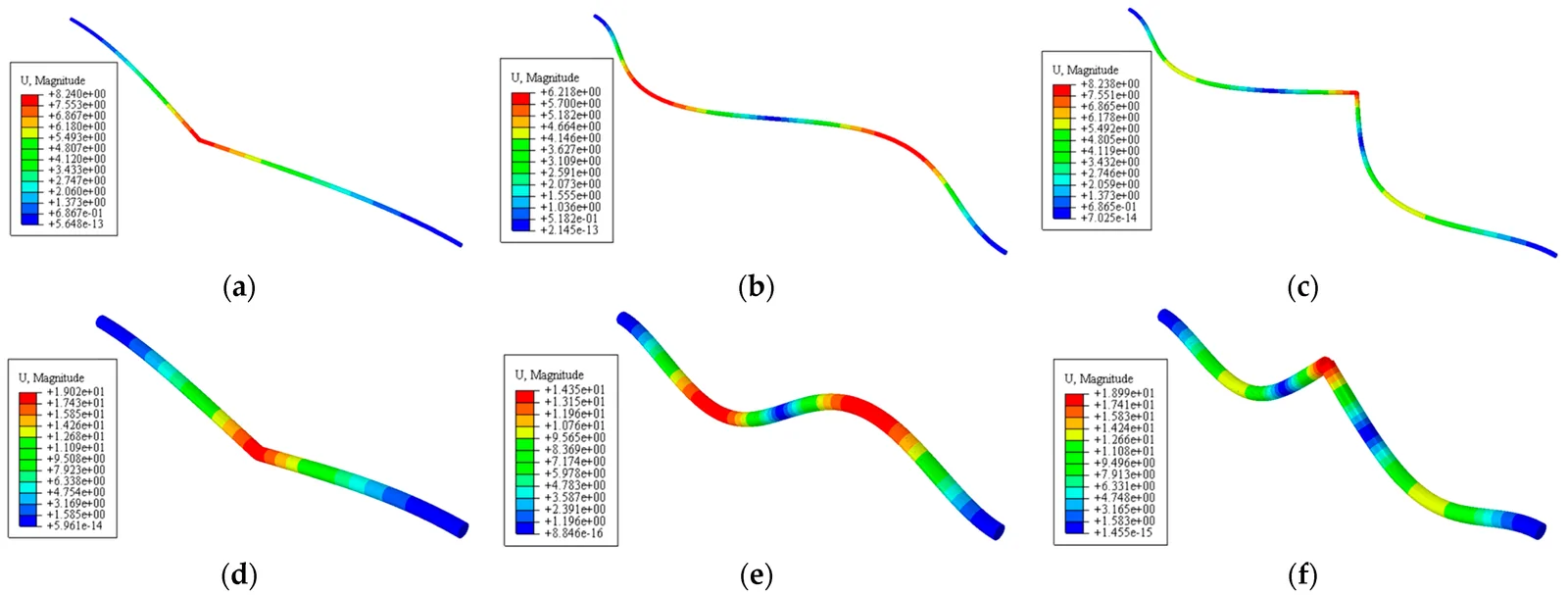
Comparison of the vibration mode shapes for Case 3-PRO and Case 3-PTFE under slider-constrained boundary conditions. (**a**) 1st system bending mode of Case 3-PRO; (**b**) 2nd system bending mode of Case 3-PRO; (**c**) 3rd system bending mode of Case 3-PRO; (**d**) 1st system bending mode of Case 3-PTFE; (**e**) 2nd system bending mode of Case 3-PTFE; (**f**) 3rd system bending mode of Case 3-PTFE.

These results demonstrate that the geometric-material distortion similarity theory proposed herein is not only independent of specific structural connection topologies but also exhibits strong adaptability to variations in external constrained boundaries. Furthermore, Figure 14 presents the system-level mode shapes of the prototype (Case 3-PRO) and the PTFE-distorted group (Case 3-PTFE) under constrained boundaries.

**Figure 15:**
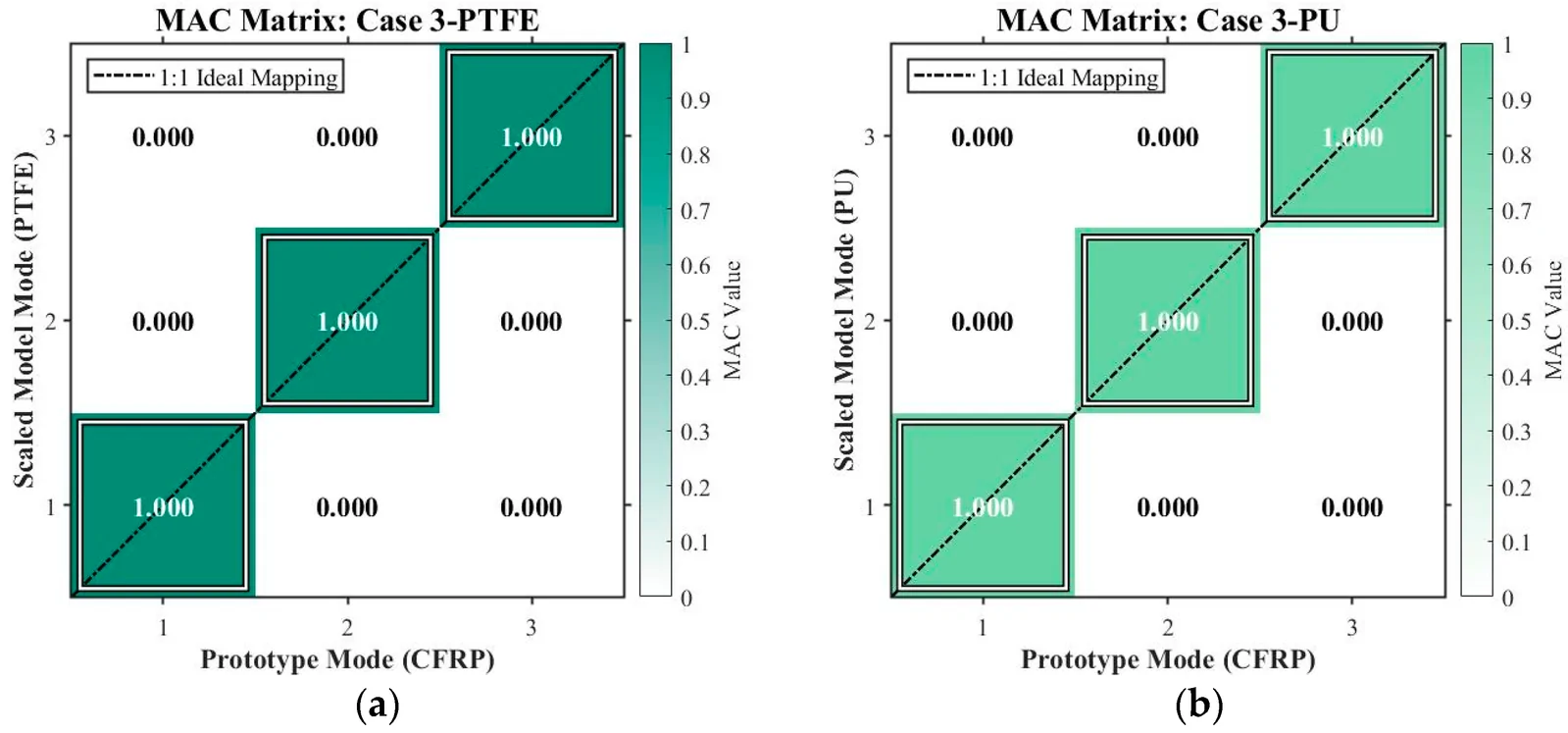
Modal Assurance Criterion (MAC) matrices compare the CFRP prototype and the scaled models. (**a**) MAC matrix for Case 3-PTFE; (**b**) MAC matrix for Case 3-PU.

Compared to the completely unconstrained results in Section 3.3, it is observable that the end constraints induce significant changes in the kinematic characteristics of the system: the first-order mode transitions from an unconstrained free bending to a constrained bow-shaped bending, and the strain characteristics at the spherical joints become more pronounced. Nevertheless, the comparison in Figure 14 clearly reveals that, despite a fundamental shift in boundary operating conditions, the distorted model is still capable of reconstructing the spatial waveform profiles and antinode distributions of the prototype system with exceptional accuracy. Furthermore, Figure 15 extracts the system-level MAC matrices of the prototype and the two distorted models under constrained boundaries. The results show that the main diagonal elements for Case 3-PTFE and Case 3-PU remain robustly around 1.000, and the off-diagonal regions show no signs of modal aliasing. This indicates that the local stiffness reconstruction induced by the distortion parameters possesses exceptional adaptability and robustness against strong external boundary constraints.

In conclusion, the validation work conducted in Section 3.4 establishes the scientific validity of the geometric-material distortion similarity theory under complex constrained boundaries. Ultimately, through progressive validation involving single-rod components, completely unconstrained multibody systems, and constrained multibody systems, the universal applicability of the proposed theory for addressing the dynamic problems of large-scale space structures characterized by cross-scale dimensions, variable topologies, and variable boundaries is comprehensively demonstrated.

### 3.5. Equivalence Validation of the Solid Scaled Model for the Hollow Prototype

As stated in Section 2.4, authentic aerospace structures predominantly use carbon fiber thin-walled or thick-walled hollow tubular components. This section further investigates whether the proposed solid equivalence theory can bridge the cross-sectional discrepancy to achieve dynamic equivalence from a hollow prototype to a solid scaled model.

In space trusses with large length-to-diameter ratios, the global low-frequency dynamic behavior of the system is primarily dominated by the bending stiffness (EI) and linear density (ρA), and is relatively insensitive to the cross-sectional shape. Therefore, during the scaling design, the hollow tube can be modeled as an equivalent solid rod simply by mapping the aforementioned physical quantities of the cross-section. The prototype in this section utilized a thick-walled CFRP hollow tube with an outer diameter of 50 mm and an inner diameter of 25 mm. To maintain the consistency of the validation system and examine the theory’s capability to handle “geometric distortion”, the solid scaled model adopted PTFE material, and its diameter was forcibly locked at 50 mm. The specific material parameters are detailed in Table 5.

In this section, numerical validations were sequentially conducted on three configurations: the unconstrained single rod, the unconstrained two-bar mechanism, and the constrained two-bar mechanism. The boundary conditions for each configuration were kept strictly consistent with those described in Section 3.2, Section 3.3 and Section 3.4. Table 6 presents a quantitative comparison of the first three bending frequencies under various operating conditions, and the visual mapping trends of the absolute frequencies and prediction errors are illustrated in Figure 13.

**Figure 16:**
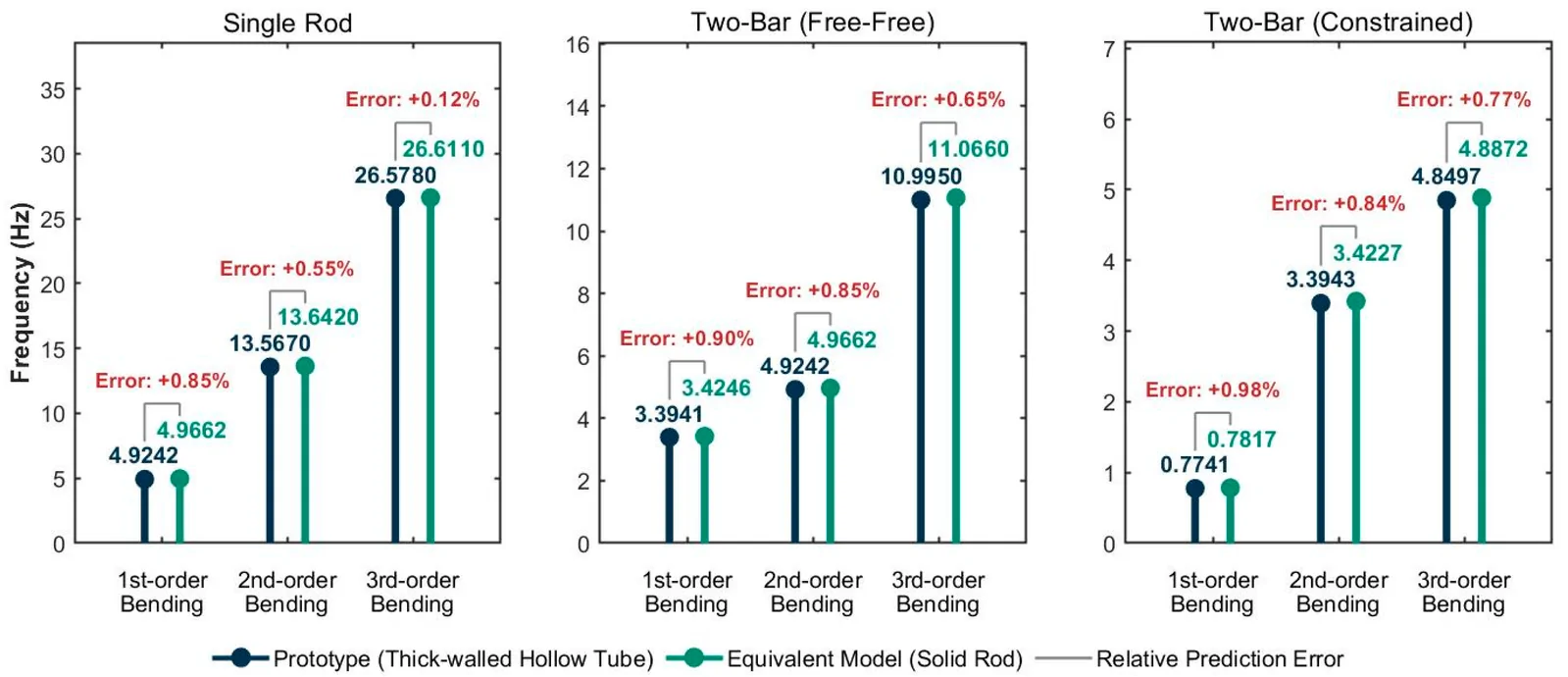
Comparison of natural frequencies and relative prediction errors between the hollow prototype and the solid equivalent model across three successive configurations.

As can be seen from Table 6 and Figure 16, whether for the single-rod basic unit or the complex multibody system connected by spatial joints, the solid equivalent model can reproduce the dynamic fundamental frequencies of the hollow prototype with high precision. The system prediction errors for all modal orders are within 1.0%. This minor discrepancy primarily stems from the inherent physical differences in the transverse shear coefficients between the hollow and solid cross-sections. Given that the low-frequency behavior of the structure is dominated by the bending stiffness EI, the influence of this shear deviation is marginal and does not result in nonlinear accumulation as the topological complexity of the system increases.

The above results confirm that the proposed distorted similarity theory can effectively overcome the limitations of cross-sectional morphology and enables the dynamic prediction of the thick-walled hollow prototype using a solid distorted model. This provides a reliable theoretical basis for high-fidelity ground scaling tests of complex aerospace truss structures.

## 4. Evaluation of Equivalent Performance and Conclusions of the Geometric-Material Distortion Similarity Theory

The numerical validation results in Section 3 demonstrated the effectiveness of the geometric distortion and material compensation similarity theory in the dynamic scaling equivalence of large-scale structures. Traditional similarity theories focus on maintaining a strict geometric profile of the structure, which is universally applicable in conventional mechanical tests. However, the results of this study showed that when dealing with large-span flexible space structures, forcefully maintaining macroscopic and microscopic geometric similarity inadvertently results in dynamic distortion due to size reduction. Through specific operating conditions, this study confirmed that permitting non-proportional size or structural distortion of the cross-section, supplemented by corresponding material substitution, is an effective method to re-establish the dynamic consistency between the prototype and the scaled model. The proposed method provides a new theoretical basis for breaking through the limitations of ground scaling tests for large-scale flexible space structures.

Furthermore, this study revealed the stability of the distortion theory under complex connections and constrained boundaries. Whether involving the topological integration across spatial spherical joints or introducing sliding rail constraints that fundamentally alter the modal configuration of the system, the prediction accuracy of the equivalent model consistently remains at an extremely high level and even exhibits an error convergence phenomenon under strongly constrained states. Analyzing this phenomenon from the perspective of structural dynamics, its core lies in the fact that the design of the distortion parameters directly reconstructs the underlying strain energy and kinetic energy distributions of the basic units. When sliding rail constraints are imposed on the system boundaries, the overall rigid body displacement is suppressed, and the deformation energy is increasingly confined to the local elastic bending of the units. This “boundary independence” demonstrates that in future ground tests, researchers can confidently introduce supporting fixtures such as guide rails and suspension slings according to site conditions to counteract the gravity effects on the ground, without the concern that external constraints will disrupt the inherent dynamic similarity relationship of the scaled model.

Despite achieving the aforementioned theoretical and numerical results, the current limitation of this study is its reliance solely on numerical validations, without physical laboratory experiments. On the one hand, direct 1g ground testing of a continuous flexible rod reaching 10,000 mm in length faces severe physical obstacles. Under a ground environment, such an enormous structural span will inevitably experience significant self-weight deflection. This self-weight introduces geometric nonlinear stiffening effects into the stiffness matrix, severely distorting the dynamic output results. Therefore, 1g ground tests cannot accurately characterize the free-floating microgravity modal behavior that this study aims to explore. On the other hand, since the core equivalent compensation law of this theory is derived under the Euler–Bernoulli assumption, it is necessary to evaluate its applicable scope through Timoshenko beam theory. The relative frequency truncation error caused by shear deformation and rotary inertia can be approximated as $\Delta f/f\approx C_{n}(1+E/\kappa G)(D/L{)}^{2}$ (where $C_{n}$ is a coefficient related to the modal order). This analytical baseline quantitatively proves that the distortion error is inversely proportional to the square of the slenderness ratio (L/D). By substituting the polymer parameters and the third-order bending mode characteristics of the free-free state into this analytical baseline, the inverse solution reveals that to strictly envelope the theoretical shear truncation errors of the first three primary frequencies within 1%, the condition L/D≥40 must be satisfied. Breaching this critical threshold will result in a nonlinear amplification of prediction errors.

To systematically address the aforementioned experimental limitations, subsequent research will design a constant-tension suspension offloading testbed to simulate the on-orbit zero-gravity environment, enabling physical testing of the polymer models. It should be pointed out that the current dynamic equivalent analysis in this paper is mainly established upon the ideal linear elastic assumption. Considering that real on-orbit assembled modules will inevitably be subjected to the coupled influences of numerous factors, such as CFRP orthotropy, the nonlinear constitutive behaviors of polymers, and non-ideal joint stiffness involving friction and clearances, caution must be exercised when extending the relevant conclusions to complex engineering systems. Therefore, as a critical cross-validation step transitioning towards physical tests, the next phase of work will construct high-fidelity Shell/Solid element finite element models that incorporate the aforementioned complex physical features. Building upon this, the Absolute Nodal Coordinate Formulation (ANCF) and flexible cable networks will be further integrated to explore the extension mechanism of nonlinear distortion similarity under large deformation conditions, aiming to comprehensively reveal the macroscopic similarity evolution laws of ultra-large space structures.

Synthesizing the aforementioned theoretical discussions and engineering prospects, the specific conclusions of this paper are summarized as follows:

(1) In terms of the scaling methodology, this paper systematically investigates the dynamic equivalence problem of the on-orbit assembly of extremely large space structures, and establishes a distortion compensation scheme between high-modulus prototypes and low-modulus scaled models. This scheme effectively overcomes the physical distortion of traditional geometric similarity in slender flexible structures, breaks through the physical limitations of hollow and solid cross-sectional shapes, and achieves high-fidelity dynamic analysis and validation across structural connections and variable boundary conditions.

(2) Regarding the prediction of dynamic characteristics, traditional complete geometric scaling leads to substantial dynamic prediction errors in large-span flexible structures. By means of the geometric distortion and material compensation similarity principle formulated in this paper, the impacts induced by macroscopic size reduction were successfully eliminated, and the exceptionally high feasibility of the cross-sectional dimensionality reduction mapping from complex thick-walled hollow tubes to equivalent solid rods was confirmed. This theory not only stably controlled the errors of the first three system bending frequencies of free single rods and completely unconstrained multibody assembly systems to within 1% but also simultaneously reproduced the spatial mode shape trends of the prototype system.

(3) Concerning complex constrained operating conditions, the distortion equivalent model exhibited exceedingly strong boundary adaptability. When sliding rail boundaries were introduced to constrain the endpoint degrees of freedom and reconstruct the global stiffness matrix of the system, the scaled model still accurately predicted the dynamic response of the prototype. The prediction errors for the system’s primary bending frequencies under the constrained state further converged to within 0.3%, and the mode shapes consistently and precisely mapped the constrained modal characteristics as well as the local strain distributions of the prototype.

This research provides a fundamental theoretical framework for space-ground dynamic consistency and ground experiments of ultra-large on-orbit assembled space structures, and offers an engineering reference for subsequent high-fidelity physical model updating and actual physical testing.

## Figures and Tables

**Figure 1:**
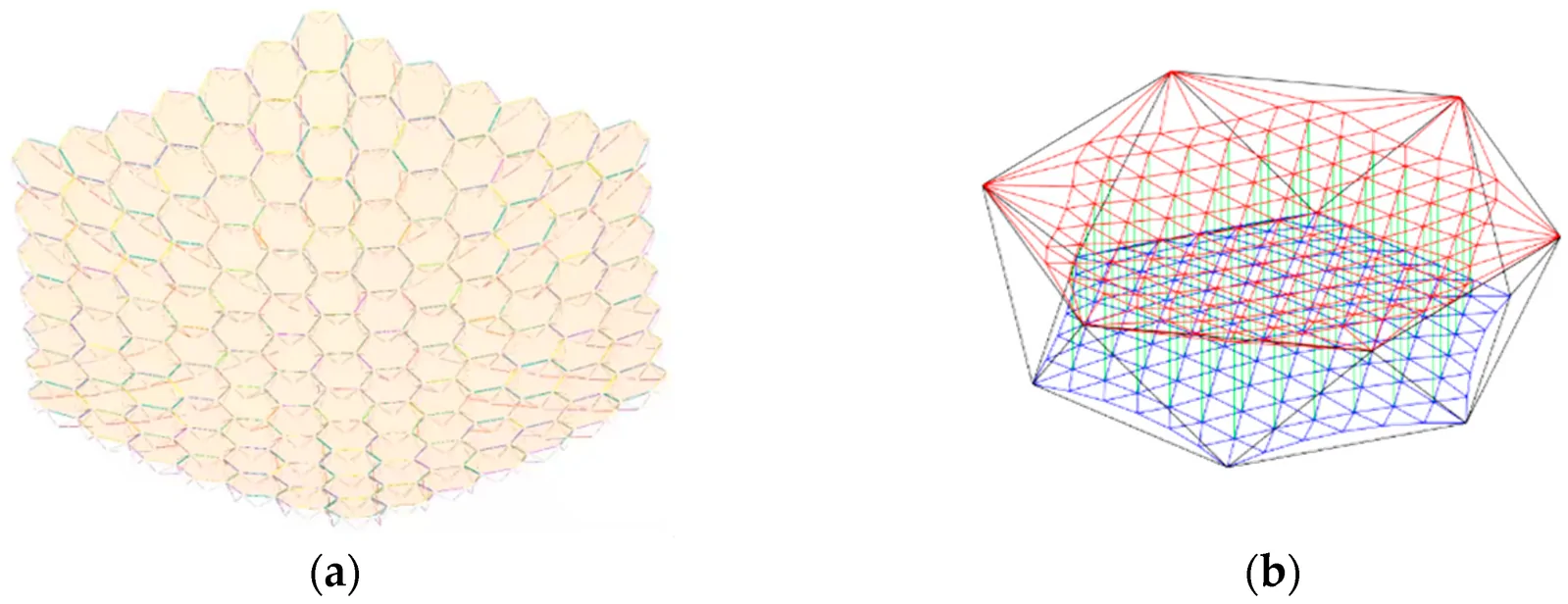
Structural configurations of the ultra-large parabolic antenna: (**a**) global modular array; (**b**) fundamental modular unit.

**Table 1:** Geometric dimensions and material properties of the single-rod model.

Test Case	Model Description	Length L (mm)	Diameter D (mm)	Elastic Modulus E (MPa):	Density ρ (ton/mm^3^)	Poisson’s Ratio ν
Case 1-PRO	Prototype (CFRP)	10,000	50	120,000	1.5 × 10^−9^	0.30
Case 1-SIM	Similar Model (CFRP)	2000	10	120,000	1.5 × 10^−9^	0.30
Case 1-PTFE	Distorted Model (PTFE)	2000	40	440	2.2 × 10^−9^	0.46
Case 1-PU	Distorted Model (PU)	2000	50	160	1.25 × 10^−9^	0.49

**Table 2:** Comparison of the natural frequencies of a single rod under different scaling and distortion strategies (free-free boundary condition).

Test Case	Model/Parameter	1st-Order Bending	2nd-Order Bending	3rd-Order Bending
Case 1-PRO	CFRP Prototype $f_{p}$ (Hz)	3.9797	10.967	21.492
Case 1-SIM	CFRP Similar Model $f_{m1}$ (Hz)	19.898	54.834	107.46
	Deviation (%)	+399.987%	+399.991%	+400.000%
Case 1-PTFE	PTFE Distorted Model $f_{m2}$ (Hz)	3.9755	10.933	21.364
	Error (%)	−0.106%	−0.310%	−0.596%
Case 1-PU	PU Distorted Model $f_{m3}$ (Hz)	3.9729	10.913	21.286
	Error (%)	−0.171%	−0.492%	−0.958%

**Table 3:** System-level natural frequency comparison of the two-bar mechanism under different scaling and distortion strategies (free-free boundary conditions).

Test Case	Model/Parameter	1st-Order Bending	2nd-Order Bending	3rd-Order Bending
Case 2-PRO	CFRP Prototype $f_{p}$ (Hz)	2.7431	3.9797	8.8877
Case 2-SIM	CFRP Similar Model $f_{m1}$ (Hz)	13.751	19.898	44.439
	Deviation (%)	+401.294%	+400.000%	+400.006%
Case 2-PTFE	PTFE Distorted Model $f_{m2}$ (Hz)	2.7410	3.9755	8.8655
	Error (%)	−0.077%	−0.106%	−0.250%
Case 2-PU	PU Distorted Model $f_{m3}$ (Hz)	2.7397	3.9729	8.8519
	Error (%)	−0.124%	−0.171%	−0.403%

**Table 4:** System-level natural frequency comparison of the two-bar mechanism under different scaling and distortion strategies (slider-constrained boundary conditions).

Test Case	Model/Parameter	1st-Order Bending	2nd-Order Bending	3rd-Order Bending
Case 3-PRO	CFRP Prototype $f_{p}$ (Hz)	0.62561	2.7434	3.9200
Case 3-PTFE	PTFE Distorted Model $f_{m2}$ (Hz)	0.62546	2.7401	3.9135
	Error (%)	−0.024%	−0.120%	−0.166%
Case 3-PU	PU Distorted Model $f_{m3}$ (Hz)	0.62537	2.7381	3.9095
	Error (%)	−0.038%	−0.193%	−0.268%

**Table 5:** Geometric dimensions and material properties of the hollow prototype and its solid equivalent model.

Model Description	Length L (mm)	Diameter D (mm)	Elastic Modulus E (MPa):	Density ρ (tonne/mm^3^)	Poisson’s Ratio ν
Hollow Prototype (CFRP)	10,000	Outer 50/Inner 25	120,000	1.5 × 10^−9^	0.30
Solid Equivalent Model (PTFE)	2000	50	440	2.2 × 10^−9^	0.46

**Table 6:** Frequency prediction errors of the solid-equivalent model relative to the hollow prototype for three configurations.

Test Case	System Configuration	Model/Parameter	1st-Order Bending	2nd-Order Bending	3rd-Order Bending
Case 4-SR	Single Rod (Free-Free)	Hollow Prototype $f_{p}$ (Hz)	4.9242	13.5670	26.5780
		Solid Equivalent $f_{m}$ (Hz)	4.9662	13.6420	26.6110
		Error (%)	+0.85%	+0.55%	+0.12%
Case 4-TBF	Two-Bar (Free-Free)	Hollow Prototype $f_{p}$ (Hz)	3.3941	4.9242	10.9950
		Solid Equivalent $f_{m}$ (Hz)	3.4246	4.9662	11.0660
		Error (%)	+0.90%	+0.85%	+0.65%
Case 4-TBC	Two-Bar (Constrained)	Hollow Prototype $f_{p}$ (Hz)	0.7741	3.3943	4.8497
		Solid Equivalent $f_{m}$ (Hz)	0.7817	3.4227	4.8872
		Error (%)	+0.98%	+0.84%	+0.77%

## Data Availability

The original contributions presented in this study are included in the article. Further inquiries can be directed to the corresponding author.
